# New Titanium Alloys, Promising Materials for Medical Devices

**DOI:** 10.3390/ma14205934

**Published:** 2021-10-09

**Authors:** Madalina Simona Baltatu, Petrica Vizureanu, Andrei Victor Sandu, Nestor Florido-Suarez, Mircea Vicentiu Saceleanu, Julia Claudia Mirza-Rosca

**Affiliations:** 1 Department of Technologies and Equipments for Materials Processing, Faculty of Materials Science and Engineering, Gheorghe Asachi Technical University of Iaşi, Blvd. Mangeron, No. 51, 700050 Iasi, Romania; cercel.msimona@yahoo.com (M.S.B.); sav@tuiasi.ro (A.V.S.); 2 Romanian Inventors Forum, Iasi. Sf. P. Movila 3, 700089 Iasi, Romania; 3 Mechanical Engineering Department, Las Palmas de Gran Canaria University, 35017 Tafira, Spain; nestor.florido@ulpgc.es; 4 Neurosurgery Department, Faculty of Medicine, Lucian Blaga University, 550024 Sibiu, Romania; vicentiu.saceleanu@gmail.com

**Keywords:** Ti–Mo–Si alloys, biomedical alloys, corrosion resistance

## Abstract

Titanium alloys are used in medical devices due to their mechanical properties, but also for their corrosion resistance. The natural passivation of titanium-based biomaterials, on the surface of which a dense and coherent film of nanometric thickness is formed, composed mainly of TiO_2_, determines an apparent bioactivity of them. In this paper, the method of obtaining new Ti20MoxSi alloys (x = 0.0, 0.5, 0.75, and 1.0) is presented, their microstructure is analyzed, and their electrochemical responses in Ringer´s solution were systematically investigated by linear polarization, cyclic potential dynamic polarization, and electrochemical impedance spectroscopy (EIS). The alloys corrosion resistance is high, and no evidence of localized breakdown of the passive layer was observed. There is no regularity determined by the composition of the alloys, in terms of corrosion resistance, but it seems that the most resistant is Ti20Mo1.0Si.

## 1. Introduction

Globally, implantology aims to use materials with specific biological and biomechanical characteristics, as high as possible [1], and titanium alloys have advantages both in terms of reducing risks to patients during and after medical interventions, and in their efficacy and biocompatibility with human tissue [2].

The concern of researchers and physicians in recent years, for the improvement of biomaterials, comes from the reason that biomaterials have to be tolerable by the organism [3] for a significant period and, therefore, have to satisfy the performance requisites in accordance with the medical purposes in which they are destined to be used [4,5].

Implant materials are produced with a high degree of complexity [6], with biological, mechanical and technological characteristics that are specific to the field of application, which must comply with strict quality standards, so as not to affect the health of patients [7,8]. Biomaterials must have special characteristics, including excellent fatigue strength, tensile strength, high corrosion and wear resistance, low modulus of elasticity, good hardness, low density, and high biocompatibility [9].

Titanium and titanium alloys, due to their properties, tend to replace the classic materials, such as stainless steels, Co–Cr alloys, etc., in most orthopedic applications [10,11]. Titanium and its alloys have prevailed because they have the optimal characteristics required for implant materials, namely, very good corrosion resistance, biocompatibility, excellent mechanical and fatigue resistance properties, toughness, low modulus of elasticity, satisfactory strength at wear, and affordable price [12].

Titanium is a non-toxic element, including in high amounts; several investigations have shown the effect of the ingesting of up to 0.8 mg of Ti by humans per day, thus demonstrating that Ti was excreted with no absorption/assimilation [13]. Many studies have shown that titanium implants do not show rejection due to their biocompatibility, good interaction with the host bone, and high resistance to corrosion [10]. An important factor in the mechanical and structural properties depends on the alloying elements added to the titanium and the appropriate design of the alloys (type of element and concentration), which can lead to them being an attractive option for medical applications.

Taking all these into account, for the design of these new alloys, we have chosen two elements, Mo and Si, due to the following considerations:

Molybdenum is an element that is widely used as an alloying element of titanium [14,15]. Some research in the field [16,17,18] has shown that a percentage of about 20% Mo can reduce the modulus of elasticity, as it is a β-stabilizing element, leading to proper mechanical properties, similar to human bone [5]. It is also a biocompatible element, with a minor level of toxicity, with respect to Cr, Ni, and Co. There is a study [19] of two titanium alloys, Ti10Mo and Ti20Mo, highlighting the formation of equiaxial beta grains of different sizes, highlighting that an alloy with a percentage of 10% Mo has grains that are larger than an alloy with a concentration of 20% Mo.

Silicon is a biocompatible element, a β-stabilizing element for titanium alloys, that reduces ductility, improves resistance to creep, and increases corrosion resistance and turnability. Jiang et al. [20] studied the corrosion resistance of Ti–Si alloys in acid solutions. Research has shown that it exhibits a high resistance to corrosion, due to the passive films formed on Ti–Si alloys, composed of TiO_2_/SiO_2_ oxides [21,22].

Even Ti–Mo alloys have received extensive attention, including the FDA certification (Food and Drug Administration–USA); however, there are not corrosion studies on the newly obtained alloys from the Ti–Mo–Si system. In this paper, the method of obtaining these new Ti20MoxSi alloys (x = 0.0, 0.5, 0.75, and 1.0) is presented, their microstructure is analyzed, and their electrochemical responses in Ringer´s solution were systematically investigated by linear polarization, cyclic potential dynamic polarization, and electrochemical impedance spectroscopy (EIS).

## 2. Materials and Methods

### 2.1. Obtaining Ti–Mo–Si Alloys

Obtaining the Ti–Mo–Si base alloys was performed by vacuum arc remelting (VAR), in MRF ABJ 900 equipment (Materials Research Furnaces, Suncook NH, USA). The raw materials used were high-purity elements such as Ti (99% purity), Mo (99% purity) and Si (99% purity) supplied by Alfa Aesar by Thermo Fisher Scientific. 

The solidification structure of the ingots resulting in VAR technology depends on the solidification rate and the temperature gradient at the liquid/solid interface. In order to avoid obtaining the primary dendritic structure, it is necessary to maintain a constant temperature gradient during the entire remelting process. The direction of growth of the cellular structure, according to the direction of the thermal gradient and the maintenance of the direction of heat dissipation in the recrystallization crucible, maintains a constant crystallization front.

There are several conditions that must be met to avoid the formation of defects in ingots, they are as follows:

- Avoidance of uniform and incomplete melting, especially of the areas enriched in alloying elements from the inter-dendritic space of the electrode subjected to re-melting;
- The spring is not strong enough to be able to dissolve and melt the alloy in the melting electrode uniformly, so recrystallization of the melt does not take place before the melting is homogenized;
- The solidification front after remelting should be uniform and at a constant speed.

The design and production of metallic materials frequently used as biomaterials requires a detailed study, both in terms of design and the technological parameters of the development equipment.

The design of the Ti–Mo–Si alloys was based on information about the physical properties of the alloying elements (melting temperature and density), as well as the equilibrium diagrams for the Ti–Mo–Si alloys [23,24,25]. 

The technical parameters regarding the vacuum arc remelting furnace used are the following: melting power—min. 55 kVA; melting current—min. 650 A, 60% DS, three-phase voltage; maximum temperature—3700 °C; continuous monitoring of the vacuum level with display of its value; maximum vacuum level obtained with preliminary and diffusion vacuum pumps: 10^−6^ mbar; inert gas supply system—argon; the oven chamber is made of 306 L stainless steel, and the double walls are cooled with water; water-cooled copper base plate measuring 230 mm (diameter) × 13 mm (thickness); non-consumable electrode made of tungsten with 6.5 mm diameter. 

The loading of the raw material in the installation was performed in ascending order of their specific density, as follows: tantalum, molybdenum and silicone, followed by the sealing closure of the chamber of the processing furnace. During the melting operations, a vacuum atmosphere of 4.5 × 10^−3^ mbar was created, followed by purging the chamber with inert gas (Ar), a cycle repeated three times to remove the air from the working chamber of the installation. The development of the process of elaboration of Ti–Mo–Si alloys was controlled through the observation window of the furnace, properly illuminated. The elaboration of Ti–Mo–Si alloys made with the help of the vacuum spring melting installation took place by melting the elements, followed by melting the alloy six times, an operation necessary in order to refine and homogenize the alloys. The melting of the elements occurred in a uniform manner, resulting in alloys with a defined and uniform chemical composition.

The amount of metallic load for experimental alloys is shown in Table 1 and its weight is approximately 30 g per alloy. The obtained Ti–Mo–Si ingots were then cut into spherical samples with a diameter of 10 mm and a thickness of 2 mm for specific tests. 

### 2.2. Microstructure and Hardness

To examine the microstructure of the Ti–Mo–Si alloys by optical microscopy, their surface was etched with Kroll reagent containing HF, HNO3 and water in a 2:1:17 volumetric ratio. Surface observations were performed with an OLYMPUS PME 3-ADL microscope. Scanning electron microscope (SEM) observations were performed using an Apreo field emission scanning electron microscope SLoVac (Thermo Fisher Scientific, Co., Waltham, MA, USA) coupled with a Bruker EDAX detector. To ensure the best imaging and analysis conditions in high vacuum, the microscope was set to operate at a voltage of 20 kV and a beam current of 1.6 nA, with a 10.0 mm working distance.

X-ray diffraction experiments were carried out with the use of a Panalytical X’Pert Pro MPD empirical diffractometer (Malvern Panalytical Ltd, Malvern, UK). As in previous studies [26], the instrument worked with a Cu Kα anode at a power of 45 kV and 40 mA in Bragg–Brentano geometry. The specimens were rotated while the data were obtained to guarantee better information capture. The purchased patterns were generated to extract the presence of the crystalline phase, lattice configuration and grain shape with the help of Malvern Panalytical HighScore Plus software.

Samples from Ti–Mo–Si system were analyzed using Wilson Wolpert 751 N (Instron Deutschland GmbH), in order to evaluate the Vickers hardness, providing an average value from eight measured points.

### 2.3. Electrochemical Tests 

All measurements were carried out in Grifols Ringer’s solution from Laboratorios Grifols, Barcelona, Spain, with the following components in mmol/l: Na^+^ 129.9; K^+^ 5.4; Ca^2+^ 1.8; Cl^−^ 111.7; and C_3_H_5_O_3_^−^ 27.2. This is a physiological solution that was modified, in which part of the Na^+^ is substituted by Ca^2+^ and K^+^ and part of the Cl^−^ by C_3_H_5_O_3_^−^. The lactate ions are converted into bicarbonate ions, which allows the pH of the solution to be regulated.

The solution was naturally aerated, tests were performed at 25 °C and the surface of alloys was prepared suitably. 

Electrochemical techniques were applied in order to characterize the metallic surfaces, and corrosion studies were performed with the following two types of potentiostats: VoltaLab PGP 201 potentiostat and PGZ 301 dynamic potentiostat. 

The VoltaLab PGP 201 potentiostat (VolataLab 21) (Radiometer Analytical SAS - France), a compact potentiostat/galvanostat, is an ideal device for electrochemical corrosion studies. This can be operated in the following two ways: with VoltaMaster 4 data collection and processing software or it can be controlled manually.

The VoltaLab PGP 201 Potentiostat was employed for the following:

- To obtain anodic polarization curves in conditions of low electrode potential sweep rates, curves that were then employed to measure the polarization resistance, the corrosion current and the corrosion rate.
- To obtain cyclic polarization curves with potential sweep rates of 10 mV/s, from which the kind of corrosion and the corrosion rate when the alloy´s potential is far from the corrosion potential were evaluated.

For both the potentiodynamic measurements and the electrochemical impedance spectroscopy determinations, a three-electrode cell was used.

The electrodes used (working electrode) were built in the form of discs (10 mm in diameter and 2 mm thick), being mounted in the cell by means of a screw and a Teflon sealing gasket. Through this mount, the surface of the electrode exposed to the corrosion medium was flat and circular, without edges or tips. The surface exposed to corrosion media was in all cases equal to 0.283 cm^2^. The samples were polished on SiC paper up to 2000 grit and then degreasing and washing with distilled water were performed. A standard 3-electrode electrochemical cell was used with Pt as auxiliary electrode and a SCE as reference electrode.

The working conditions used in the measurements were as follows:

- Linear anodic polarization: potential range (−200) ÷ (+500) mV vs. SCE, potential scanning speed—dE/dt = 0.5 mV/s;
- Cyclic polarization: potential range (−500) ÷ (+2000) mV vs. SCE, potential scanning speed = 10 mV/s;
- EIS measurements: working potential = open circuit potential, frequency range = 100 kHz ÷ 100 mHz, AC potential amplitude of 10 mV and single sine wave measurements were conducted.

All potentials were measured against the saturated calomel electrode. 

Open circuit potential, E_OCP_ (I = 0), was measured, and Tafel slopes (b_a_ and b_c_), polarization resistance (R_p_), corrosion current density (J_corr_) were calculated using the facilities offered by VoltaMaster 4 software. For smoothing the curve, 9 points were taken into account. The calculation area was ±120 mV from E_corr_ and the linear segment was 30 mV on each of the two branches of the polarization curve (cathodic line and anodic line).

In all studied cases, when interpreting the linear polarization data, a calculation area of 120 mV and a linearity segment of 30 mV were considered.

Electrochemical impedance spectroscopy (EIS) data were used, which were acquired with VoltaMaster 4 software and processed with ZSimpWin software, in which the electrochemical spectra are interpreted by the least squares method developed by Boukamp. For this purpose, VoltaMaster data were converted to ZSimpWin processable data with EIS file converter software (Radiometer Analytical S.A.).

## 3. Results

### 3.1. Microstructure and Hardness

The elemental compositions obtained by energy-dispersive X-ray spectroscopy analysis (EDX) for the Ti–Mo–Si alloys present no impurities in the metallic mass, and Ti, Mo, and Si were the main elements identified. Table 2 present the average results of ten EDX points of the Ti–Mo–Si alloys surfaces.

**Figure 1:**
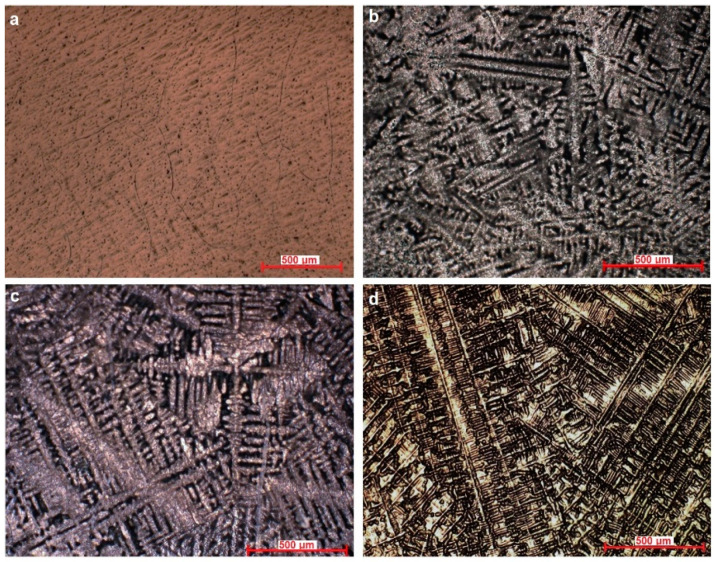
Optical microstructure of the alloys at 100X. (**a**) Ti20Mo, (**b**) Ti20Mo0.5Si, (**c**) Ti20Mo0.75Si, (**d**) Ti20Mo1.0Si.

The microstructure of the Ti–Mo–Si alloys was highlighted by optical microscopy, see Figure 1. Ti20Mo has a structure with a β crystal grain, and Ti20Mo0.5Si, Ti20Mo0.75Si, and Ti20Mo1.0Si show a dendritic structure. 

**Figure 2:**
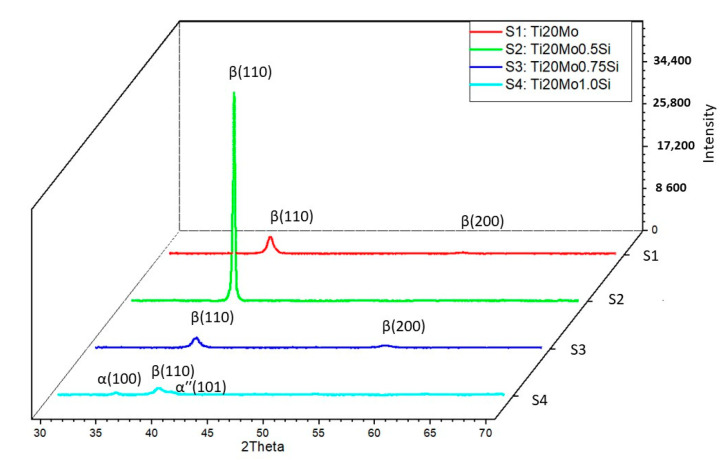
XRD patterns Ti–Mo–Si alloys: S1—Ti20Mo, S2—Ti20Mo0.5Si, S3—Ti20Mo0.75Si, S4—Ti20Mo1.0Si.

In addition to the structure of the Ti–Mo–Si alloys, the X-ray diffraction (XRD) was also highlighted, see Figure 2. The effect of the addition of Si can be clearly observed in both Figure 1 and Figure 2.

All the alloys were mainly composed of the parent β phase, with different precipitates of minor secondary phases, such as the α′′ martensite phase and α phase. 

Using the Mo–Si and Mo–Ti binary diagrams, the following three stable compounds were determined in these alloys: Ti4.00Si8.00 (reference code: 96-100-9013), Ti4.00 (reference code: 96-901-1601), and Ti2.00 (reference code: 96-901-2925). These compounds that were formed in alloys were identified by XRD analysis. The main characteristics for Ti4.00Si8.00 are as follows: crystal system: orthorhombic, a (Å): 4.4280, b (Å): 4.7790, c (Å): 9.0780, alpha (°): 90.00, beta (°): 90.00, gamma (°): 90.00, calculated density (g/cm^3^): 3.60, and volume of cell (106 pm3): 192.10. The main characteristics for Ti4.00 are as follows: crystal system: hexagonal, a(Å): 2.9700, b (Å): 2.9700, c (Å): 4.7200, alpha (°): 90.00, beta (°): 90.00, gamma (°): 120.0000, calculated density (g/cm^3^): 8.82, and volume of cell (106 pm^3^): 36.06. The main characteristics for Ti2.00 are as follows: crystal system: cubic, a (Å): 3.2820, b (Å): 3.2820, c (Å): 3.2820, alpha (°): 90.00, beta (°): 90.00, gamma (°): 90.00, calculated density (g/cm^3^): 4.50, and volume of cell (106 pm^3^): 35.35. 

The β Ti2.00 phase, which has a body-centered cubic structure, is obtained by alloying titanium with transition elements (Mo, Si), while the αʹʹ (HC) phase, Ti4.00Si8.00, is a martensite, crystallized in the orthorhombic system, and is generated by increasing the amount of stabilizing elements in the β phase, which decomposes during the cooling process.

The hardness measurement results from the Ti–Mo–Si samples are as follows: Ti20Mo: 409HV, Ti20Mo0.5Si: 348 HV, Ti20Mo0.75Si: 170 HV, and Ti20Mo1.0Si: 175HV. Accordingly, with the obtained results, the addition of Si to Ti–Mo alloys significantly decreases the values of hardness. Compared to other biomaterials, Ti20Mo0.75Si alloy is close to the 316 L stainless steel alloy (155HV) value.

### 3.2. Linear Potentiodynamic Polarization

The corrosion potential, E_corr_, is a measure of the corrosion tendency of a metal or alloy immersed in an electrolytic medium. High negative values of this parameter indicate a high probability that the metal will corrode in that environment. This value was measured directly versus a saturated calomel electrode (Hg/Hg_2_Cl_2_/KCl sat.), after 15 min of thermostating the sample in the corrosion medium. The measured parameter represents the open circuit potential (OCP), and its value should be equal to the corrosion potential (E_corr_) only after the metal/electrolyte balance is reached. The time after which this equilibrium is reached depends on the temperature and the processes that take place at the metal–solution interface (chemical reaction, adsorption of some species from the solution, passivation or dissolution). Several preliminary tests have indicated that this time is of the order of several hours.

For this reason, the corrosion potential was evaluated indirectly from the linear polarization curves, using the Evans diagram, which represents the logarithm of the current density as a function of the electrode potential in a range of ±50 mV. In these coordinates, the intersection of the linear portions of the anodic and cathodic branches of the polarization curve gives the value of the corrosion potential on the axis, E_corr_ (Evans).

The polarization resistance method was used to evaluate the corrosion rate. This method serves to determine the corrosion current, at the corrosion potential of the metal or alloy, from the linear polarization curve obtained for a relatively small overvoltage. The corrosion current determined by this method therefore represents the current that appears at the metal/corrosive medium interface when the metal is immersed in the solution, and represents the instantaneous corrosion current. From a practical point of view, the density of the instantaneous corrosion current is important, (J_corr_ = I_corr_/S), which is directly correlated with the corrosion rate (v_p_), according to Equations (1)–(4).

(1) $$R_{p}={\left(\frac{dE}{dj}\right)}_{\left(E_{corr}\right)}=\frac{b_{a}\cdot b_{c}}{2.303\cdot J_{corr}\cdot \left(b_{a}+b_{c}\right)}\left[ohm.cm^{2}\right]$$

(2) $$J_{corr}=\frac{b_{a}+b_{c}}{2.303\left(b_{a}+b_{c}\right)\cdot R_{p}}\left[\mu A/cm^{2}\right]$$

(3) $$v_{p}=3.27\cdot \left(\frac{A}{z}\right)\cdot \frac{J_{corr}}{\rho }\left[\mu m/year\right]$$

where the following applies:

(4) $$b_{a}={\left(\frac{\partial E}{\partial logj}\right)}_{a}\;and\;b_{c}={\left(\frac{\partial E}{\partial logj}\right)}_{c}\left[mV/dec\right]$$

**Figure 3:**
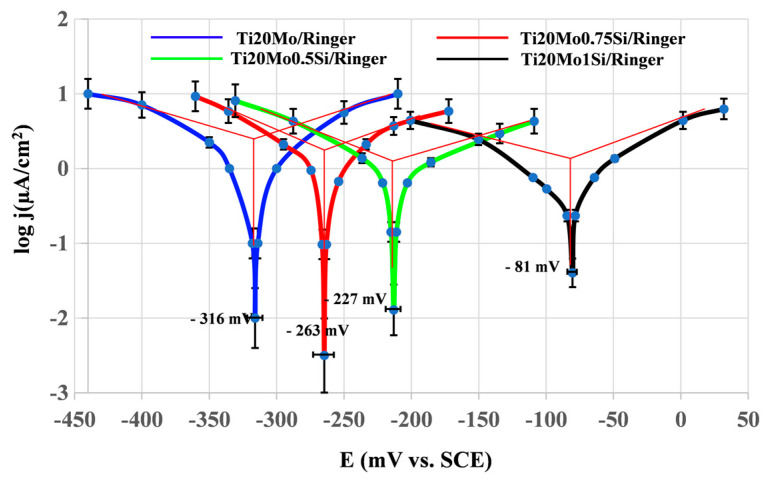
Linear polarization curves in semi-logarithmic coordinates for Ti–Mo–Si alloys in Ringer’s solution.

The linear polarization curves, in semilogarithmic coordinates (Evans diagram), for the tested specimens in Ringer’s solution are displayed in Figure 3, and Table 3 shows the instantaneous corrosion parameters in this physiological environment.

The presence of silicon produces a decrease in the corrosion rate.

### 3.3. Cyclic Potentiodynamic Polarization

One of the methods for further characterization of the corrosion process is cyclic potentiodynamic polarization, and additional information on the processes taking place in the system can be obtained. 

In the present study, the cyclic polarization curves (cyclic voltammograms) were recorded in the potential range (−500…+2000…−500) mV vs. SCE, with a potential sweep rate of 10 mV/s. All the measurements were made in naturally aerated solutions. It started from a sufficiently high negative potential, in order to reduce all ionic or molecular species that contaminate the surface of the alloy. The speed of variation in the working electrode potential was relatively high (10 mV/s), in order to obtain current intensities that were high enough to cover possible accidental fluctuations in the system, but low enough to detect all the processes that could occur in the solution or on the surface of the electrode.

Based on these curves, the following characteristic parameters of the passivation process were evaluated: the corrosion potential (E_corr_), the repassivation potential (E_rp_), and, in some cases, the potential for transpassivation (E_tp_). In addition, in order to be able to make a comparison between the process intensities at the various alloy/corrosion medium pairs, the current densities corresponding to the maximum applied overpotential, respectively, 2V (J_2V_), were evaluated. For the calculations, cyclic voltammograms were used both in linear coordinates, j = f (E), and in semi-logarithmic coordinates, E = f (log j). From the linear diagram, J_2V_ and E_tp_ were evaluated, while, from the semi-logarithmic diagram, E_corr_ and E_rp_ were evaluated.

At potentials higher than the E_tp_ potential on the oxide-coated surface, transpassivation phenomena occur, which, in this case, consist of the release of oxygen.

An additional use of cyclic voltammograms was made, in order to evaluate the corrosion rate (passivation) based on the anodic polarization curve obtained at a high potential scanning speed (10 mV/s). For this purpose, the Tafel method (polarization resistance method), applied for the points on the anodic branch of the polarization curve located in the vicinity of the corrosion potential (±120 mV compared to E_corr_), was used. The following were evaluated: polarization resistance (R_p_), corrosion current density (J_corr_), and corrosion rate (v_corr_). 

**Figure 4:**
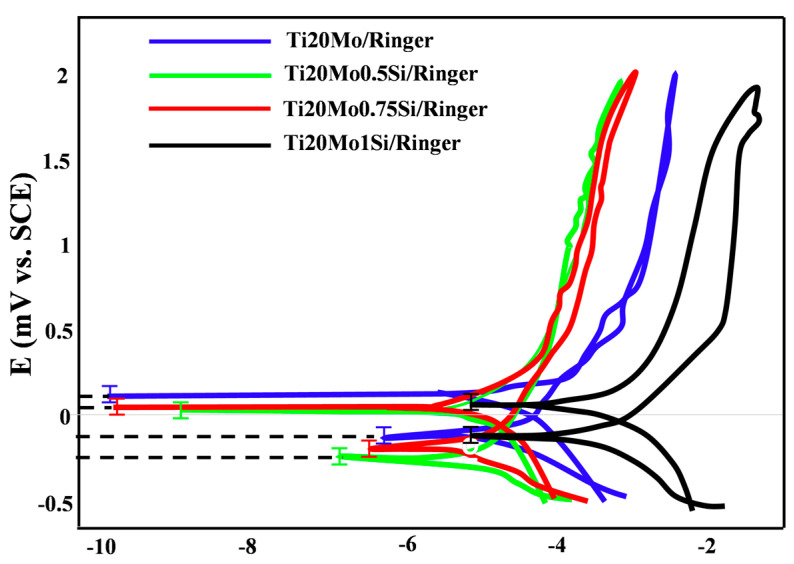
Corrosion parameters obtained from cyclic polarization curves in Ringer’s solution.

The values of the characteristic potentials and the corrosion parameters evaluated in the case of using a sweep rate of 10 mV/s, obtained for the alloys in the corrosion media, are presented in Table 4, and the cyclic voltammograms in semi-logarithmic coordinates are presented in Figure 4. 

For most samples, the anodic curve (branch of the cyclic voltammogram obtained by scanning the potential from negative values to positive values, −0.5 V → 2 V) is located above the cathode curve (return branch, +2 V → −0.5 V), and this indicates a passivation of the alloy. As a result of this passivation, the repassivation potentials are more positive than the corrosion potentials, but no correlation can be established between E_corr_ and E_rp_, and no rule of dependence of the two potentials on the composition of the alloy can be established. 

### 3.4. Electrochemical Impedance Spectroscopy

Using the ZSimWin program, the experimental data obtained with the PGZ 301 potentiostat were converted using the “EIS file converter” software (EISFC150).

**Figure 5:**
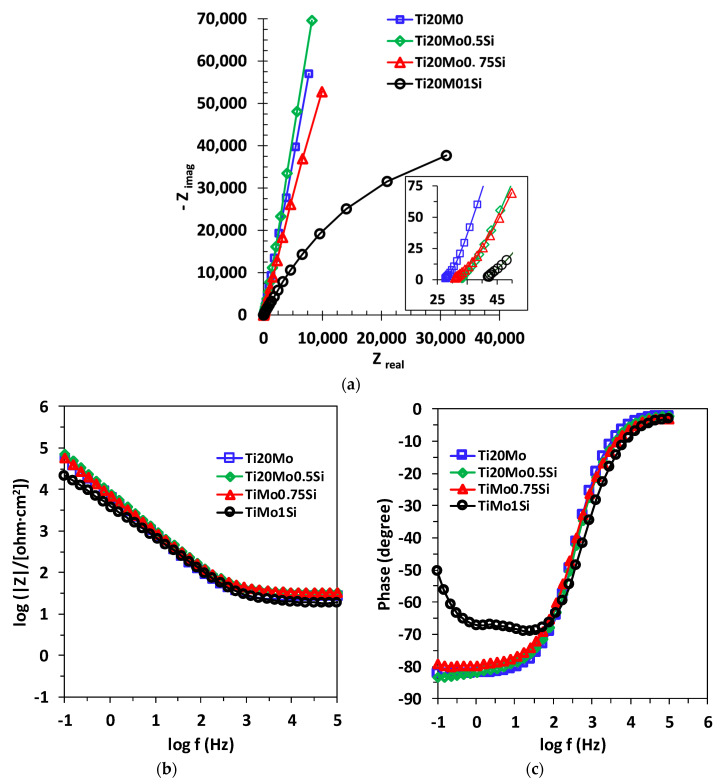
(**a**) Nyquist,(**b**) Bode—IZI and (**c**) Bode—phase diagrams for Ti20Mo, Ti20Mo0.5Si, Ti20Mo0.75Si and Ti20Mo1.0Si alloys in Ringer’s solution.

The EIS data were represented in the Nyquist diagram (imaginary impedance component as a function of the real impedance component: –Z_im_ = f (Z_real_)) and in the Bode diagram. An example of the Nyquist and Bode diagrams corresponding to the experimental data series obtained for the alloys studied in Ringer’s solution is shown in Figure 5. The experimental data can be fitted with different equivalent circuits that are associated with the physical model of the surface.

**Figure 6:**
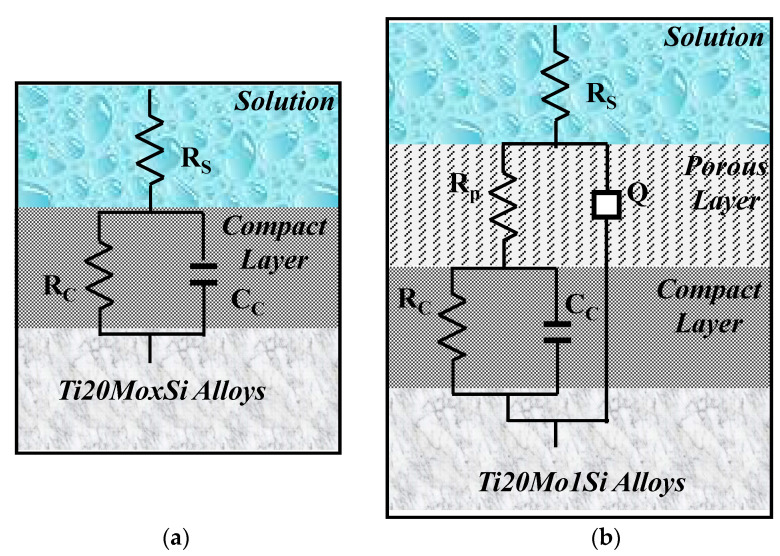
The equivalent circuit and the solution–alloy interface for the following: (**a**) Ti20Mo, Ti20Mo0.5Si, Ti20Mo0.75 Si; (**b**) Ti20Mo1.0Si.

The obtained spectra were interpreted by modeling the data with the equivalent circuits shown in Figure 6. The shape of the Bode spectra for the Ti20Mo1.0Si alloy is clearly different in comparison with the other alloys, suggesting a two-layer structure of the solution–alloy interface, as illustrated in Figure 6b. 

The fitting results were consistent with the experimental data, and the chi-squared values were of the order 10^−3^, see Table 5, which demonstrated that good simulation quality was achieved. As shown in the table, comparing Ti20Mo with Ti20Mo0.5Si, it can be observed that with the rise in Si content, the value of R_C_ increased, while the values of C decreased, suggesting that the corrosion resistance of the Ti20Mo alloy is improved due to the addition of Si.

Equivalent circuits that are associated with this physical model have also been proposed for anodized aluminum [27] and for passive films on titanium alloys [28,29,30].

In this equivalent circuit, R_S_ represents the resistance of the solution between the sample and the reference electrode, R_P_—the resistance of the porous layer, and R_C_—the resistance of the compact layer. In this case, in order to widen the scope of application of the model, instead of the ideal capacitance, a constant phase element (CPE) was introduced, the impedance of which is defined by the following relation:

(5) $$Z_{CPE}=\frac{1}{Y_{0}{\left(j\omega \right)}^{n}}$$

where Q is a particular combination of circuit elements, which cannot be separated in the electrochemical system, mainly related to surface inhomogeneity and solid-phase mass transport. The introduction of such an element is recommended when, in the Nyquist diagram, the semicircle is open and incomplete, ω is the angular frequency, and j is the imaginary number (j^2^ = −1). The exponent n is related to the slope of the linear portion in the log frequency – IZI graph of the Bode diagram and can take values between –1 and +1. If the exponent n = 0, CPE is an ideal resistor, while if n = 1, then CPE is an ideal capacitor. If the exponent n has the particular value n = ½ at high frequencies, the circuit element is called the diffusive Warburg impedance. Even if a phase constant element was used in this study, it was assimilated with a non-ideal capacitor (C) when 0.8 < n < 1, and with a Warburg impedance when 0.5 < n < 0.65.

## 4. Discussion

### 4.1. Linear Potentiodynamic Polarization

With the exception of two alloys, the passivation rates are of the same order of magnitude, with the lowest passivation rate being presented by the Ti20Mo1.0Si alloy. 

In terms of quantity, the corrosion potentials of the Ti–Mo–Si samples studied are negative and relatively small, between 80 and 300 mV in Ringer’s solution. The differences between the corrosion potentials of these samples (below 250 mV) are not significant; such differences may occur as a result of the difference in surface processing, contamination of the surface with organic substances, different aeration of the corrosion medium, temperature variations, etc.

The corrosion rates (actually passivation rates) are of the same order of magnitude, relatively small (15–30 µm/year), for all the alloys. It seems that the most stable alloy is Ti20Mo1.0Si.

The potentiodynamic analysis indicated that the addition of silicon minimized the anodic and cathodic reactions, thus reducing the corrosion current densities up to almost 50% (see Table 3). Also, the addition of silicon affected the displacement of the corrosion potential values to more positive values, with respect to the absence of Si, suggesting that the addition of silicon had a greater effect on the anodic reaction than on the cathodic reaction, by forming SiO_2_ in the passive film. The Si–O bond is a strong covalent bond, and is helpful to achieve the loss mid-gap states and passivate the surface of the alloy.

In Ringer´s solution, the corrosion rate values also follow the same trend as the corrosion current density, with the values decreasing due to silicon addition. It is very important to report that the polarization resistance (Rp) values rise with the increase in Si concentration, recording a max. value of 21.55 kΩ·cm^2^_._

### 4.2. Cyclic Potentiodynamic Polarization

The study of the behavior of alloys at high potentials, by recording cyclic voltammograms at large potential domains, gives information on the behavior of alloys when they are immersed for a longer period of time.

The maximum current densities (corresponding to E = 2V) are very small, below 1 mA/cm^2^, and the vast majority are below 0.5 mA/cm^2^. Accordingly, the corrosion rates are equally low at 15–55 μm/year in Ringer’s solution. 

There is no regularity determined by the composition of the alloys, in terms of corrosion resistance, but it seems that the most resistant is Ti20Mo1.0Si.

In Ringer´s solution (see Table 4), we can observe that without Si, the alloy has a bigger corrosion current density than TiMoxSi alloys, and this can be due to the fact that the SiO_2_ serves as a barrier against corrosion.

### 4.3. Electrochemical Impedance Spectroscopy

The shape of the alloys curves for the electrochemical impedance spectra indicates that all the alloys studied have similar behavior in Ringer’s solution, with small differences depending on the composition of the alloy. The Nyquist curves, obtained at E_corr_, indicate a capacitive behavior, characterized by incomplete semicircles, while the Bode representations show one or two constants for the relaxation time. 

The obtained spectra, interpreted by modeling the data with an equivalent circuit with two time constants, suggest a two-layer structure of the passive film. 

The fitting results were consistent with the experimental data, and the chi-squared values were of the order 10^−3^ (see Table 5), which demonstrated that good simulation quality was achieved. As the tables show, the addition of Si affects both the characteristics of the external porous passive layer (process dominated by ion diffusion) and the internal compact layer (process dominated by charge transfer).

There are no appreciable variations in the values of ohmic resistance of the solution, which means that no ions were released in the solution during the experiment. The values of R_C_ are higher than the R_P_ values, reflecting that the outer porous film exhibits lower resistance than the inner barrier layer.

It can be observed that the fitted values of C_C_ decrease with the addition of Si, indicating that the thickness and stability of the protective passive film increased due to SiO_2_ formation.

## 5. Conclusions

Four new alloys from the Ti–Mo–Si system were obtained by vacuum arc remelting, and the corrosion behavior of these alloys was studied. 

The analysis of corrosion performance was carried out by electrochemical methods (linear potentiodynamic polarization, cyclic potentiodynamic polarization, and electrochemical impedance spectroscopy) in artificial (simulated) physiological environments. 

The polarization resistances show very low corrosion rates. The process taking place on the surface of all the alloys is the oxidation of titanium, with the development of an oxide layer on the surface (TiO_2_), and Si additions improve the corrosion resistance of the alloys.

Cyclic potentiodynamic polarization revealed that there is no regularity determined by the composition of the alloys, in terms of corrosion resistance, and the most resistant alloy was Ti20Mo1.0Si.

The obtained EIS spectra show a compact passive layer for Ti20Mo, Ti20Mo0.5Si, and Ti20Mo0.75Si, and a two-layer structure of the solution/alloy interface (one porous and another compact passive film for Ti20Mo1.0Si).

It can be concluded that the silicon addition minimized the anodic and cathodic reactions, thus reducing the corrosion current densities up to almost 50% (see Table 3). Also, the addition of silicon affected the displacement of the corrosion potential values to more positive values, with respect to the absence of Si, suggesting that the addition of silicon had a greater effect on the anodic reaction than on the cathodic reaction, by forming SiO_2_ in the passive film, which acted as a barrier against corrosion.

All the alloys have shown chemical stability and high corrosion resistance, and, from a chemical point of view, can be recommended to be used for future medical applications.

## Figures and Tables

**Table 1:** Calculation of the metallic load for the experimental alloys.

Alloy		Element [g]			Batch Weight [g]	Ingot Weight [g]	Efficiency [%]
		Ti	Mo	Si			
S1	Ti20Mo	24.01	6.01	-	30.02	29.97	99.83
S2	Ti20Mo0.5Si	23.88	6.03	0.14	30.05	30.00	99.83
S3	Ti20Mo0.75Si	23.70	6.12	0.22	30.04	30.01	99.90
S4	Ti20Mo1.0Si	23.70	6.05	0.31	30.06	30.03	99.90

**Table 2:** Average elemental compositions.

Sample		Ti [wt.%]	Mo [wt.%]	Si [wt.%]
S1	Average	80.05	19.95	-
	Stdev	±0.2	±0.1	-
S2	Average	80.15	19.44	0.41
	Stdev	±0.1	±0.1	±0.01
S3	Average	79.74	19.55	0.71
	Stdev	±0.3	±0.2	±0.02
S4	Average	78.98	20.01	1.01
	Stdev	±0.5	±0.3	±0.01

**Table 3:** Instantaneous corrosion parameters for Ti–Mo–Si alloys in Ringer’s solution.

Alloys	E_corr_ [mV vs. SCE]	R_p_ [kohm.cm^2^]	J_corr_ [µA/cm^2^]	V_corr_ [µm/year]	b_a_ [mV/dec]	b_c_ [mV/dec]
Ti20Mo	−315	13.84	2.71	26.25	192	−205
Ti20Mo0.5Si	−227	17.71	2.09	20.19	310	−142
Ti20Mo0.75Si	−263	16.50	2.68	25.89	289	−219
Ti20Mo1.0Si	−81	21.55	1.61	15.55	168	−224

**Table 4:** Corrosion parameters obtained from cyclic polarization curves in Ringer’s solution.

Alloys	E_corr_ [mV vs. SCE]	E_rp_ [mV]	E_tp_ [mV]	J_2V_ [mA/cm^2^]	Tafel Parameters on v_s_ = 10 mV/s		
					R_p_ [KΩ.cm^2^]	J_corr_ [µA/cm^2^]	V_corr_ [µm/Year]
Ti20Mo	−124	+112	1704	1.28	6.14	5.583	53.96
Ti20Mo0.5Si	−235	+42	1070	0.46	9.41	3.788	36.59
Ti20Mo0.75Si	−183	+52	1500	0.59	10.68	3.424	33.09
Ti20Mo1.0Si	−77	+100	-	0.23	22.06	1.624	15.69

**Table 5:** The values of the circuit for Ti20Mo, Ti20Mo0.5Si, Ti20Mo0.75 Si and Ti20Mo1.0Si alloys in Ringer solution.

Alloys	Circuit	R_s_ [Ω.cm^2^]	10^5^ × C_C_ [Ss^n^/cm^2^]	R_C_ [Ω.cm^2^]	10^5^ × Y_0_ [F/cm^2^]	n_1_	R_P_ [Ω.cm^2^]	10^3^ × χ^2^
Ti20Mo	R(CR)	30.47	1.85	2.53 · 10^5^	-	-	-	38.4
Ti20Mo0.5Si	R(CR)	37.93	1.45	3.40 · 10^5^	-	-	-	53.3
Ti20Mo0.75Si	R(CR)	39.22	1.61	3.89 · 10^5^	-	-	-	71.0
Ti20Mo1.0Si	R(Q(R(CR)))	18.88	1.07	0.39 · 10^5^	4.75	0.817	1.81 · 10^4^	0.63
